# Vesicoureteral Reflux, a Scarred kidney, and Minimal Proteinuria: An Unusual Cause of Adult Secondary Hypertension

**DOI:** 10.1155/2011/913839

**Published:** 2011-11-09

**Authors:** Shaifali Sandal, Apurv Khanna

**Affiliations:** Department of Medicine, SUNY Upstate Medical University, 343 CWB, 750 E. Adams, Syracuse, NY 13210, USA

## Abstract

Hypertension affects about 65 million individuals in the United States. In adult patients, primary aldosteronism and renovascular causes are described as most prevalent. Vesicoureteral reflux as a cause of hypertension, while commonly described in pediatric populations, is less prevalent in the adult population especially in the absence of proteinuria. We present the case of a 31-year-old female presenting with early onset hypertension. Workup for renovascular hypertension was unrevealing. She was found to have right-sided vesicoureteral reflux with a unilateral scarred kidney. Patient underwent a nephrectomy with marked improvement in blood pressure control.

## 1. Introduction

The absolute number and percentage of adults with hypertension was estimated to be at least 65 million and 31.3%, respectively, in the US from years 1999-2000 [1]. In the adult population, 5–10% cases of hypertension are identified to be due to secondary causes such as renal parenchymal disease from diabetes or hypertension, fibromuscular dysplasia, atherosclerotic disease, primary aldosteronism, pheochromocytoma, Cushing's disease, hypo/hyperthyroidism, hyperparathyroidism, and obstructive sleep apnea [2]. 

Vesicoureteral reflux (VUR) is a less recognized cause of hypertension in the adult population. It is a congenital anomaly of the ureterovesical junction due to deficiency of the submucosal longitudinal muscle that predisposes the individual to retrograde urine reflux. Adult presentation of primary VUR occurs in patients who have had VUR since childhood that has remained undetected and presents in adulthood with complications or patients who develop VUR in adulthood de novo with no prior history of UTI [3]. Primary VUR occurs in the absence of any underlying pathology, while secondary VUR in the adult occurs due to conditions such as obstruction, previous surgical procedures, or a neurological disorder [3]. Long-term sequalae include end-stage renal disease, proteinuria, hypertension, urinary tract infections, and renal calculi [3–5]. Incidence of hypertension in adults with reflux nephropathy has been reported to be anywhere between 38–60% [4–9] often associated with proteinuria. We present a case of a 31-year-old female who had right-sided VUR with a scarred right kidney as the cause of her underlying hypertension.

## 2. Case Presentation

A 31-year-old female with onset of hypertension in adolescence initially presented to us with a recent history of hypertensive urgency and prior history of systolic blood pressure of 220 mm of Hg. She had been started on Amlodipine 10 mg once daily by her primary care physician and referred to the University Nephrology Clinic. Past medical history was significant for workup for secondary hypertension through a local nephrology practice approximately nine years ago. At that time, renal artery stenosis was considered the most likely diagnosis and was pursued with invasive testing. She underwent a renal arteriogram at that time which did not reveal stenosis. Careful history by us, however, revealed recurrent urinary tract infections and back pain accompanying these episodes which aroused our suspicion for a diagnosis of pyelonephritis and vesicoureteral reflux. 

Her family history was significant for middle age onset of primary hypertension in her father; her mother was diagnosed with autosomal dominant polycystic kidney disease leading to end-stage renal disease. She was sexually active with no use of oral contraceptives. On presentation to us, her physical examination revealed blood pressure of 153/97 mmHg, equal in both arms, and with strong pulses in upper and lower extremities. The remainder of the physical examination was essentially unremarkable; no femoral, carotid, or abdominal bruits were appreciated. 

Urinalysis revealed 2+ leukocyte esterase, positive for nitrites, trace protein, trace blood, and white blood cell count of 47. Her complete blood count and basic metabolic panel were unremarkable. Serum creatinine was 0.7 mg/dL, and 24-hour urine protein was 0.24 gm/24 hours (<0.2 gm/24 hours). 

Ultrasound of the kidneys showed a right kidney measuring 7.8 cm with severe cortical thinning and the left kidney measuring 13.2 cm with normal cortical echogenicity. There was no evidence of hydronephrosis or renal calculi. Real-time sonographic, duplex, and color Doppler images of the kidneys demonstrated a scarred right kidney. Doppler of renal arteries did not show renal artery stenosis. A voiding cystourethrogram demonstrated grade-3, right vesicoureteral reflux. There was also focal narrowing of the right ureterovesical junction. The bladder was within normal limits. Nuclear imaging to evaluate renal flow showed prompt radiopharmaceutical uptake within the left kidney. The right kidney was not clearly visualized on flow phase or functional images. On delayed imaging, there was reflux into the right collecting system. 

She was started on hydrochlorothiazide 25 mg once daily by mouth. Angiotensin converting enzyme inhibitors were not used due to the patient being sexually active without use of contraception. Subsequently, surgical opinion was sought regarding possible nephrectomy. The patient underwent a laparoscopic right nephrectomy and partial ureterectomy successfully. Microscopic analysis of the kidney revealed chronic pyelonephritis and extensive cortical atrophy. At four month followup, the patient demonstrated excellent blood pressure control at 118/88 mm of Hg while maintained on hydrochlorothiazide 12.5 mg once daily by mouth.

## 3. Discussion

### 3.1. VUR and Hypertension in Children

Secondary hypertension is more common in the pediatric population. 60–80% of secondary hypertension is caused by renal parenchymal disease [10]. VUR, multicystic dysplastic kidney, ureteropelvic junction obstruction, and posterior urethral valves have been associated with hypertension [11]. VUR is one of the most common causes of severe hypertension in children [6, 10–13]. 

VUR was noted in 29% to 50% of children with UTIs, often the initial presentation [14]. The prevalence of hypertension in children with primary vesicoureteral reflux was estimated to increase with age: it was 1.7% for patients aged 1 year to 9.9 years, 1.8% for adolescents aged 10 years to 14.9 years, 4.7% for patients aged 15 years to 19.9 years, and 35% for patients >20 years of age [15]. It was estimated that 50% of patients with unilateral and bilateral renal damage would be hypertensive at about 30 and 22 years of age, respectively [15]. Hypertension was strongly associated with renal scarring, and VUR increases renal scarring [6, 15].

### 3.2. VUR and Hypertension in Adults

Incidence of hypertension in adults with reflux nephropathy has been reported to be anywhere between 38–60% [4–9]. A retrospective study reported nearly 60% of patients with VUR had hypertension at presentation; it was significantly associated with the presence of >1 g/day of proteinuria, although the exact number of patients who had both proteinuria and hypertension was not reported [5]. Studies have reported an inherent relationship between hypertension and proteinuria in patient who have VUR [9]. This was not the case with our patient. Our patient had a 24-hour urine protein of 0.24 gm/24 hours. 

To our knowledge, only one study in adults so far has reported serious consideration of VUR as a secondary cause hypertension in the absence of renal injury markers. In a study by Barai et al., 19.1% of adult patients with hypertension who do not have any evidence of renal involvement had VUR and are often labeled as having essential hypertension [16]. An editorial did criticize this study as none of the patients had frequent UTI's, an inherent selection bias may have existed as none of the patients had an indication to have a direct radionuclide voiding cystoscintigraphy (DRVC) performed, and, lastly, that no imaging study was performed to evaluated renal scarring [17]. For our patient, we performed a real-time sonographic, duplex, and color doppler images of the kidneys which did reveal a scared right kidney.

### 3.3. Cause

In the absence of proteinuria, the underlying cause of how VUR and a scarred kidney cause hypertension is unclear. Past studies have suggested that reflux nephropathy can activate the renin angiotensin system. In children, it has been shown that patients with VUR have high circulating or selective renal vein renin levels [10]. One study suggested no consistent evidence to support the role of renin-angiotension and hypertension in patients with unilateral reflux nephropathy [18]. Another study showed upregulation of angiotensin II receptors in reflux nephropathy [19].While one study from Spain showed genetic polymorphisms of renin-angiotensin-aldosterone (RAS) components are not independent prognostic indicators of renal scarring in patients with VUR [20], another study from Taiwan showed that angiotensin-converting enzyme gene polymorphisms were associated with progressive renal deterioration in Taiwanese children with VUR [21].

### 3.4. Diagnosis

Guidelines for diagnosis of VUR in the adult population have been devised with reference to the screening for VUR in the pediatric population [3]. A recent review article stated that voiding cystourethrography is the gold standard in reflux diagnosis in the pediatric population [22]. Renal sonography is often recommended as the initial evaluation followed by micturating cystourethrogram, radionuclide cystography, intravenous pyelography, ultrasound, CT, and MRI [3]. Barai et al. did mention that, in patients who do not have any signs of renal parenchymal and renovascular injury as demonstrated by negative serum creatinine, captopril renography, and urinalysis for proteinuria, studies such as DRVC should be included as part of a workup for VUR [16]. They, however, recommended further retrospective studies prior to making DRVC as standard of care in looking for underlying causes of hypertension.

### 3.5. Treatment

Treatment options can be surgical or medical. Medical treatment consists of antimicrobial treatment or prophylaxis of UTI and treatment of hypertension; however, long-term antibiotic prophylaxis may not be an ideal option for a younger patient in whom more definitive treatment would be preferred [3]. 

Surgical options have been well studied in the pediatric population. Primary ureteral reimplantation, extravesical and intravesical surgical techniques, ureteroneocystotomy, and endoscopic subureteral injections are some of the techniques employed in the correction of VUR in the pediatric population with success rates of 92–98% [23]. In adults, some studies have reported endoscopic correction using polytetrafluoroethylene (Teflon) or dextranomer/hyaluronic acid copolymer [24], endoscopic trigonoplasty [25], or endoscopic subureteral glutaraldehyde cross-linked collagen injection [26]. 

The only two indications for surgical correction of unilateral reflux in adults are a history of pyelonephritis with unsuccessful medical treatments or in women of childbearing age [27]. Recent studies, however, indicate that pregnant women with low-grade VUR and unscarred kidneys did not have any significant complications except a higher incidence of urinary tract infection, which was not modified by ureteric reimplantation [28]. Surgery, however, has been reported to have no beneficial effect on hypertension [29]. A randomized control trial failed to support the claim that renal function is improved by surgical correction of VUR in children with bilateral disease [30]. No recommendations exist for treatment specifically for hypertension in patients with VUR.

## 4. Conclusion

Our patient's workup was significant for recurrent urinary tract infections, grade 3 vesicoureteral reflux, minimal proteinuria, and a scarred kidney. Ultrasound with Doppler and voiding cystourethrogram were used for diagnosis. Medical treatment was not an ideal option in our young patient of child bearing age. A nephrectomy was performed primarily for persistent hypertension felt to be secondary to the unilaterally scarred kidney. After nephrectomy, our patient demonstrated markedly improved blood pressure control. 

Through this case we hope to highlight VUR as a cause of secondary hypertension. Markers of renal injury such as elevated creatinine and proteinuria may be absent; however, a scarred kidney probably is the cause of patient's hypertension through an unknown mechanism. Medical management may not be ideal, and stenting is not the optimal treatment for a scarred kidney. Nephrectomy may be therapeutic in these cases. The long-term benefits remain to be seen, and randomized control trials are needed to study the efficacy of surgical versus medical management of VUR in the adult population.
